# Genomic Analysis of Low-Grade Serous Ovarian Cancer: Clinical and Biological Insights

**DOI:** 10.7759/cureus.96385

**Published:** 2025-11-08

**Authors:** Anthi-Maria Papahliou, Constantinos G Zografos, Eleni Zografos, Alkistis Papatheodoridi, Garyfalia Bletsa, Ekaterini Domali, Flora Zagouri

**Affiliations:** 1 First Department of Obstetrics and Gynecology, National and Kapodistrian University of Athens, Alexandra General Hospital, Athens, GRC; 2 First Department of Surgery, Laiko Hospital, School of Medicine, National and Kapodistrian University of Athens, Athens, GRC; 3 Oncology Unit, Department of Clinical Therapeutics, National and Kapodistrian University of Athens, Alexandra General Hospital, Athens, GRC; 4 Department of Biology, Research Center, Hellenic Anticancer Institute, Athens, GRC

**Keywords:** copy number variations, epigenetic regulation, genomic profiling, kras, low-grade serous ovarian carcinoma, mapk pathway mutations, mek inhibitors, targeted therapy

## Abstract

Low-grade serous ovarian cancer (LGSOC) is a rare and clinically unique epithelial ovarian cancer (EOC) subtype that occurs in younger women, presents in an indolent but progressively persistent fashion, and is relatively resistant to conventional chemotherapy.

LGSOC has a stable genome and low mutational load (median mutational burden <1 mutation/Mb), very different from high-grade serous ovarian cancer, which is characterized by high genomic instability, with nearly universal TP53 mutation. Its pathogenesis is strongly associated with activation of the MAPK pathway. Mutually exclusive mutations in KRAS, BRAF, or NRAS are detected in approximately 50-60% of LGSOC cases and often arise early in the development of serous borderline tumors. Further features, such as NF1 loss, MAP2K1 mutations, ERBB2 activation, and frequent copy number changes (including CDKN2A/2B deletion and 1p/1q imbalances), add to tumor heterogeneity and evolution.

In addition to genomics, transcriptional and epigenomic profiling uncovers a high prevalence of estrogen receptor signaling, epithelial-mesenchymal transition-related pathways, and promoter hypermethylation of tumor suppressors (CDH1 and RASSF1A). Additionally, loss of microRNAs (e.g., miR-7) and deregulated chromatin regulators further promote tumor cell survival and resistance to apoptosis. These layers highlight that, however "silent" the enzymatic activity has been described to be, the biology of LGSOC is driven by a complex set of molecular and regulatory mechanisms.

Clinically, identification of the dependency on MAPK has guided targeted therapies. The cooperative GOG 281/LOGS trial showed that trametinib, an MEK inhibitor (MEKi), was significantly more effective than standard-of-care options (including chemotherapy or hormonal therapy) in increasing progression-free survival (median PFS 13.0 months vs. 7.2 months; hazard ratio 0.48, p < 0.001). Nevertheless, while MEKi have demonstrated clinical benefit, the emergence of resistance (frequently associated with activation of the NOTCH pathway) highlights the need for rational combination strategies. The therapeutic horizon extends with hormonal treatment targeting ER/PR and exploratory approaches using epigenetic agents, BCL-2 inhibition, and DDR-targeted therapy.

In summary, LGSOC is biologically and clinically distinct from HGSOC and from other subtypes of ovarian cancer. Genomic and multi-omic profiling have revealed actionable vulnerabilities and precision oncology approaches. The advent of biomarker-directed trials, molecular subtyping incorporation, and innovative computational strategies is likely to gradually ameliorate therapy selection and, thereby, finally improve long-term outcomes for patients with this complex disease.

## Introduction and background

Low-grade serous ovarian cancer (LGSOC), characterized by indolent clinical behavior and poor response to conventional cytotoxic chemotherapy, represents a distinct clinicopathologic and molecular subtype of epithelial ovarian cancer (EOC) [1]. Unlike high-grade serous ovarian cancer (HGSOC), which exhibits extensive genomic instability and nearly universal TP53 mutations, LGSOC harbors a much lower mutational burden and is genetically more stable [1].

Ovarian cancer overall is a heterogeneous disease comprising several histologic subtypes with distinct molecular and clinical behaviors. LGSOC accounts for approximately 5-10% of all serous ovarian cancers and typically affects younger women. It follows an indolent but persistent clinical course, with reported five-year overall survival rates of 60-85%, though many patients experience multiple recurrences during the disease course.

Histologically, LGSOC shows well-differentiated serous architecture with minimal nuclear atypia, and it frequently arises from serous borderline tumors (SBTs), suggesting a stepwise progression model [2]. This contrasts sharply with the abrupt development of HGSOC, often from serous tubal intraepithelial carcinoma (STIC) lesions in the distal fallopian tube [2,3]. Ovarian cancer classification follows a dualistic framework that differentiates low-grade from high-grade serous carcinomas based on their distinct molecular features and biological behavior [3]. LGSOC, in particular, is primarily driven by activating mutations in the MAPK signaling pathway - most notably in the KRAS, BRAF, and NRAS genes - which are detected in more than half of all cases [1,4]. These genetic alterations - which often occur early during tumorigenesis - tend to be mutually exclusive and suggest that each one may be sufficient to drive tumor development on its own [4]. On the other hand, TP53 mutations - which are almost universal in HGSOC - are rarely observed in LGSOC, reinforcing the distinct molecular pathways that characterize these two tumor types [1].

Despite advances in molecular profiling, major knowledge gaps persist regarding prognostic biomarkers, mechanisms of resistance, and optimal treatment sequencing in LGSOC. Therefore, this review aims to synthesize current genomic and multi-omic findings, clarify the biological underpinnings of the disease, and highlight emerging therapeutic opportunities to inform precision oncology strategies.

LGSOC tends to occur in younger women and follows a slow but persistent clinical course, characterized by multiple recurrences over time [5]. Due to its limited responsiveness to conventional platinum-based chemotherapy, there is a need for more personalized treatment strategies that also reflect its specific molecular features [1,5]. This subtype of ovarian cancer is really challenging, and there is growing interest in using comprehensive genomic profiling to identify actionable targets and support more tailored therapeutic approaches for patients with LGSOC [1].

This review brings together current insights into the genomic underpinnings of LGSOC and explores how they may inform future targeted therapies and translational advances.

## Review

Material and methods

This narrative review provides an overview of the molecular landscape of LGSOC and discusses recent findings regarding its genomic features and clinical relevance. A systematic search through databases, such as PubMed, Scopus, and Web of Science, was performed in the period from January 2000 to April 2023. The search was conducted with the keywords including “low-grade serous ovarian cancer,” “genomic analysis,” “molecular profiling,” “mutations,” “BRAF,” “KRAS,” “MEK inhibitors,” and “precision oncology.” Titles and abstracts of initially screened studies. The full-text articles were subsequently obtained and read in detail to see if they met the inclusion criteria (on genomic and molecular profiling of LGSOC). Peer-reviewed journals that reported original research, systematic reviews, and clinical trial data were preferred. Non-original language papers, case reports, and preprints were excluded. The inclusion criteria were the original research studies, systematic reviews, meta-analyses, and high-quality clinical trial reports that reported molecular and genomic features of LGSOC. Only English-language publications were included.

The reference lists of selected articles were also reviewed to identify any further pertinent studies. Although this is a narrative review, the methodological quality and potential bias of the included studies were considered based on study design, sample size, and clarity of genomic data reporting. The review aimed to summarize major findings pertaining to the prevalence and clinical utility of gene mutations, as well as patterns of germline and somatic rearrangements within both LGSOC and HGSOC. Preference was for studies providing interpretative evidence of clinical or therapeutic impacts of these genomic features, in particular to support targeted treatment strategies and patient prognosis.

Data Extraction and Synthesis

For each included study, data regarding study design, molecular findings, mutational frequency, clinical relevance, and therapeutic implications were extracted and summarized. Given the narrative nature of this review, no formal statistical or meta-analytic synthesis was performed. Instead, the findings were organized thematically to reflect the genomic, transcriptomic, and epigenomic features of LGSOC and their translational significance.

Quality and Bias Assessment

Although this is a narrative review, the included studies were critically evaluated for methodological quality, reproducibility, and sample size. Preference was given to studies providing interpretative or clinical evidence supporting targeted treatment strategies and patient prognosis.

As this is a narrative review, no formal meta-analysis or statistical synthesis was conducted, and findings were qualitatively summarized.

**Figure 1:**
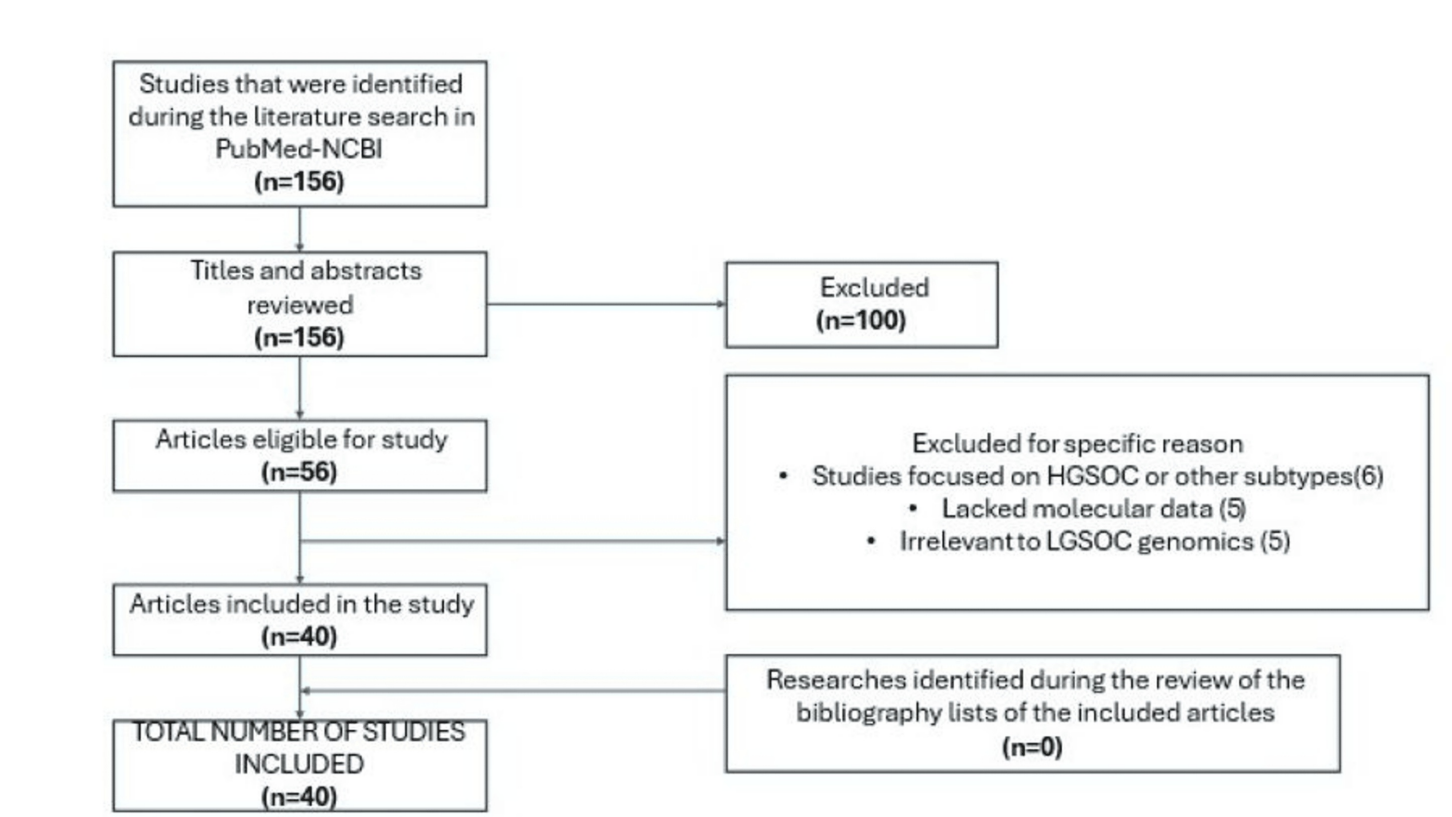
Flow chart illustrating the process of literature identification.

Figure 1 presents a flow chart depicting the process used to identify relevant literature.

Molecular landscape of LGSOC

**Figure 2:**
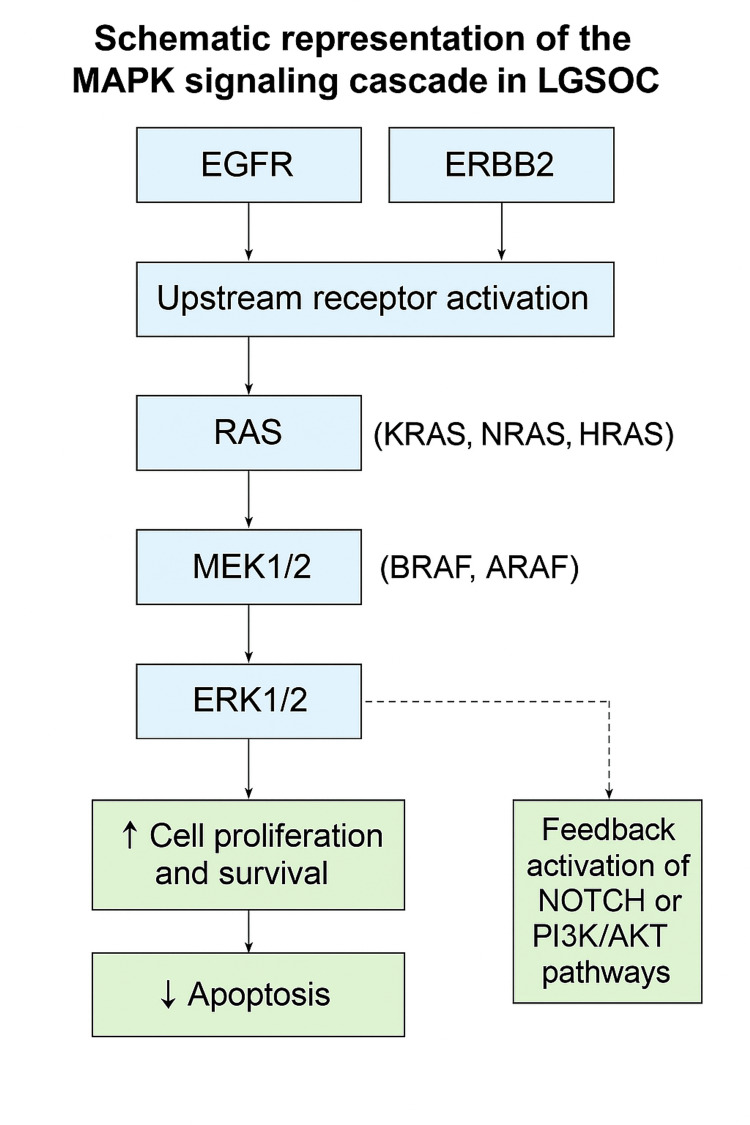
Schematic representation of the MAPK signaling cascade in low-grade serous ovarian cancer (LGSOC).

Figure 2 presents a schematic representation of the MAPK signaling cascade in LGSOC.

LGSOC is considered to have distinct genomic features that separate it from the high-grade counterpart of the same tumor type. One of its most important molecular hallmarks is the frequent activation of RAS/MAPK signaling, mainly mediated by somatic mutations involving both classical and rare RAS pathway genes [6]. These genomic alterations are not only involved in tumor initiation and development but can also be biomarkers guiding target therapy. Crucially, variance in mutational frequency among cohorts demonstrates the impact of geographic and procedural factors; this emphasizes that there should be standardization of sequencing methods.

Canonical MAPK Pathway Mutations

The most frequently mutated genes in LGSOC are KRAS, BRAF, and NRAS, all of which belong to the canonical RAS/MAPK cascade.

The KRAS mutations are the most common. They are present in ~33-50% of cases, with most of them occurring at codon 12 (e.g., G12D, G12V) and leading to constitutive activation of RAS and downstream signaling. These mutations are often clonal and appear early in tumorigenesis, frequently detected in the precursor lesion, SBTs [6,7]. Comparative analysis of KRAS-mutant versus BRAF-mutant tumors suggests distinct biological behavior and potential differences in response to MEK inhibition.

The BRAF mutations, primarily V600E, occur in about 5-20% of LGSOCs, more commonly in SBTs than in invasive carcinomas. BRAF-mutant tumors often follow an indolent clinical course and are rarely associated with progression to aggressive disease. However, some series report late relapses even in BRAF-mutant cases, cautioning against oversimplified prognostic interpretation [7,8].

The NRAS mutations are less common (~5-10%), they often affect codon 61 (e.g., Q61R), and appear to be more specific to invasive LGSOC, but are rarely found in borderline tumors. This suggests a potential role for NRAS mutations in the transition from borderline to invasive carcinoma [9, 10]. The mutual exclusivity of KRAS, BRAF, and NRAS mutations reinforces the concept that a single driver event is sufficient for MAPK activation, yet it also limits opportunities for combination targeted therapy.

Importantly, these three mutations are mutually exclusive, indicating that a single activating event in the MAPK pathway is typically sufficient for tumor initiation.

Non-canonical MAPK-Associated Mutations

Beyond the canonical genes, multiple other mutations contribute to MAPK pathway dysregulation in LGSOC:

NF1 (Neurofibromin 1), a negative regulator of RAS, is recurrently mutated or deleted in LGSOC. Loss-of-function mutations in NF1 - more frequently seen in MAPK-associated or MAPKwt subtypes - lead to unchecked RAS signaling and may contribute to de novo MAPK activation [11]. This observation highlights alternative routes to pathway activation that may explain resistance to single-agent MEKi.

MAP2K1 (MEK1) mutations have also been identified, particularly in patients with exceptional responses to MEKi. Grisham et.al reported a 15-nucleotide deletion in the negative regulatory helix of MAP2K1, resulting in constitutive ERK pathway activation and therapeutic sensitivity [12]. Such genotype-phenotype correlations provide a rationale for precision enrollment in clinical trials.

ERBB2 (HER2) activating mutations or amplifications occur in up to 6% of serous tumors of low malignant potential and LGSOC, serving as alternative RAS/MAPK activators [13].

Other rare mutations with possible MAPK relevance include alterations in FLT3, KMT2C, and TEK, which may act via crosstalk with the MAPK or PI3K pathways [14]. Integration of these low-frequency events into broader pathway models is essential for understanding therapeutic resistance.

Copy Number Alterations

While LGSOC is genomically more stable than HGSOC, recurrent copy number alterations (CNAs) have been observed. Especially, loss of chromosome 1p and gain of 1q (Chr1pq) is seen in 30-40% of cases. These CNAs correlate with worse prognosis and define a subgroup of tumors with low TMB and inferior survival [11]. Homozygous deletions at 9p21.3, targeting the CDKN2A/2B locus, are significantly more frequent in LGSOC than in SBT. In case of loss of these tumor suppressors (p16^INK4A^ and p14^ARF^), cell cycle progression and disease advancement may be facilitated [6]. These recurrent CNAs, though fewer than in HGSOC, may carry independent prognostic value and warrant incorporation into future risk-stratification models.

Other Mutated Genes

Recent large-scale exome sequencing efforts have revealed additional somatic mutations in LGSOC, many of which are novel or infrequent but may contribute to tumor evolution:

USP9X and EIF1AX, both involved in mTOR signaling and translation regulation, are recurrently mutated. Treatment resistance can be affected by these mutations, and that is why they have been proposed as potential co-drivers [6]. Mutations in genes such as FOXA1, KMT2A, and STAG2 have been identified in individual recurrent tumors and could potentially reflect intratumoral heterogeneity or molecular evolution over time [14]. For therapeutic intervention, leveraging this pathway's role in tumor maintenance can offer promising targets, especially among MAPK wild-type tumors [11]. The convergence of NOTCH activation with MAPK independence highlights a possible escape mechanism and supports investigation of combined pathway inhibition.

Overall, LGSOC is characterized by early and recurrent mutations in MAPK pathway genes-most notably KRAS, BRAF, and NRAS-along with non-canonical alterations such as NF1 and MAP2K1, deletions of tumor suppressors like CDKN2A/2B, and other less common mutations in genes including EIF1AX, USP9X, and those of the NOTCH family. This genomic heterogeneity plays a critical role in disease progression and treatment response, underscoring the need for biomarker-driven approaches to personalize therapy and improve clinical outcomes. Collectively, these data reveal a disease that, while genetically more stable than high-grade counterparts, exhibits diverse molecular vulnerabilities that can be exploited for precision oncology.

Genomic instability and copy number variations

Genomic instability is less common in LGSOC as compared to HGSOC. Yet it continues to play a prominent role in tumor progression and heterogeneity. In contrast to high-grade serous carcinomas, which have a high degree of aneuploidy and are commonly TP53-mutated, LGSOC tends toward a more limited spectrum of chromosomal aberrations. These often comprise fewer, yet recurrent CNVs. A well-known example is the hemizygous loss of the 1p36 region that harbors possible tumor suppressor genes, including miR-34a. This mutation is frequently observed in LGSOC but not in SBTs, suggesting that this alteration may be involved in progression from borderline to invasive disease [15]. Broad genome profiling has also reported frequent gains of 1q and 8q, and losses of 22q and 19q in LGSOC [16]. Notably, the recurrence of specific CNVs across independent cohorts reinforces their biological significance and reduces the likelihood that they represent random passenger events. Some of these alterations are broadly consistent with those that we and others have reported for borderline tumors, but are more common in invasive tumors, in keeping with the idea of stepwise malignant progression [16]. Interestingly, Birch et al. reported that copy number aberrations in LGSOC include losses in regions harboring PI3K and RAS pathway regulators, and that the genomic profiles of LGSOC differ significantly from those of HGSOC, despite some overlap [17]. These observations emphasize that even within the relatively “quiet” genome of LGSOC, selected structural alterations may converge on key signaling pathways and drive clinically relevant heterogeneity.

More recent integrative analyses emphasize the clinical potential of CNV data. Graf et al. demonstrated that CNV burden, even independent of BRCA status or tumor mutational load, can stratify patient prognosis in serous ovarian cancer, suggesting a possible translational application even in genomically stable subtypes like LGSOC [18]. 

Thus, despite its genomic restraint, LGSOC exhibits a reproducible pattern of CNVs that contribute to its biology and may serve as biomarkers for progression or treatment stratification. Future prospective studies should validate these CNV signatures in clinically annotated cohorts to determine their utility in guiding targeted therapy or surveillance strategies. Incorporating these CNV signatures into clinical practice could refine prognostication and guide future targeted therapeutic development [15-18].

Transcriptomic and epigenomic features

Transcriptomic and epigenomic profiling have revealed important aspects of LGSOC biology, including regulatory activities that go beyond genomic mutations. Although LGSOC harbors fewer somatic mutations than HGSOC, it shows specific dysregulation at the RNA and epigenome level [19]. Such multilayered regulation suggests that transcriptional and epigenetic changes may compensate for the relatively low mutational burden, driving tumor phenotype and therapy response.

Tan et al. found that transcriptomic analysis of LGSOC is consistent with a hybrid epithelial-mesenchymal phenotype that is enriched with respect to estrogen receptor (ER) signaling, particularly in MEK-inhibitor refractory tumors [20]. Their results also propose that EMT transcription factors, such as ZEB1 and SNAI2, might be activated differentially according to MAPK-pathway dependence, pointing at new targets for resistant LGSOC subgroups [20]. In the case of epigenetic regulation, Stružinská et al. conducted an integrated analysis of methylation and gene expression and also revealed epigenetically silenced tumor suppressor genes such as CDH1, ZIC1, and RASSF1A in LGSOC tissues [21]. Promoter hypermethylation of such key regulators highlights an actionable layer of tumor suppression loss that could be reversed by demethylating agents. These data suggest that epigenetic repression, in particular promoter hypermethylation, is involved in progression and might be involved in chemoresistance [21]. Complementing information from Swiercz et al. showed that expression of miR-7 is significantly reduced in LGSOC as compared with HGSOC and that this reduction is associated with retention of BCL2 expression and the possibility of resistance to apoptosis [22]. MicroRNA dysregulation, therefore, represents another potential therapeutic axis that is independent of DNA mutation. Moreover, Alsiary et al. identified altered expression of chromatin regulatory genes MCPH1 and ASPM, suggesting a role for dysregulated mitosis and CIN in serous carcinomas [23]. Together, these studies demonstrate that epigenetic and transcriptomic reprogramming do not represent passive events but rather active mechanisms of disease duration and drug resistance.

Collectively, the transcriptomic and epigenomic data underscore the complexity of LGSOC beyond its “quiet” genome. They reveal key regulatory crosstalk that describes the tumor phenotype and may drive therapeutic sensitivity and resistance profiles [19-23]. A key challenge will be to incorporate these layers with genomic data to enable rational combination therapies and patient outcome predictions.

Molecular distinction of lgsoc from other epithelial ovarian cancer subtypes 

LGSOC has a distinct molecular landscape that separates it from other types of EOC, such as HGSOC, mucinous, endometrioid, and clear cell carcinomas. This molecular distinction not only represents variation in biology but also plays an important role in therapy decision-making and prognostication. Recognizing these differences is critical to avoid therapeutic extrapolations from HGSOC, which may not be effective in LGSOC.

The most striking distinction, however, was in TP53 status. Mutations of TP53 are almost ubiquitous in HGSOC (~95%) but are extremely rare in LGSOC, which is uniformly TP53 wild-type [7,11]. Rechsteiner et al. showed that TP53 mutations were detected in 58.7% of high-grade serous carcinoma, 52% of clear cell, and 57% of mucinous EOC, yet were absent in LGS cases. This striking difference highlights a distinct pathogenesis [24]. The absence of TP53 disruption may partly explain the lower genomic instability and slower clinical course of LGSOC.

In the context of its driver mutations, LGSOC is largely reliant on activating mutations of the RAS/MAPK signaling cascade, in particular KRAS, BRAF, and, more rarely, NRAS. These are present in 60-70% LGSOC and its precursor SBTs but almost never in HGSOC [6,7,24]. For instance, BRAF V600E mutations, reported at frequencies up to 48% in LGSOC and SBTs, are not present in HGSOC and are infrequent in mucinous or endometrioid subtypes [13]. Conversely, mucinous carcinomas also harbor KRAS mutations at a higher frequency (up to 57%), which can often co-occur with TP53 mutations (which is not seen in LGSOC, where KRAS and TP53 mutations are mutually exclusive) [24]. Likewise, BRAF mutations are nearly absent in mucinous, clear cell, and endometrioid tumors, thereby implying that this mutation is very specific for LGSOC and S-LMP serous carcinomas. Moreover, HGSOC has extensive genomic instability and high TMB, while LGSOC has a relatively stable genome with few CNAs, low TMB, and microsatellite stability [11,14]. This genomic stability reinforces the need to focus on targeted pathway inhibition rather than DNA-damaging chemotherapies, which dominate HGSOC management.

Taken together, such molecular differences further corroborate the concept of the separate tumorigenic pathways leading to LGSOC, and it should be considered as a biologically and clinically distinct entity within the EOC classification. Failure to recognize this distinct biology risks suboptimal therapeutic choices and underscores the importance of precision oncology approaches tailored to LGSOC.

Therapeutic targets and treatment implications

MAPK Pathway and MEK Inhibition

LGSOC presents a therapeutic dilemma due to its indolent course, low mitotic index, and inherent resistance to platinum-based chemotherapy. While conventional cytotoxic regimens remain standard in initial management, their limited efficacy has catalyzed a shift toward targeted therapies that exploit the unique molecular landscape of LGSOC [25]. This shift reflects a broader recognition that traditional chemotherapy paradigms, successful in high-grade serous carcinoma, cannot simply be extrapolated to LGSOC without loss of efficacy.

A defining feature of LGSOC is its frequent activation of the MAPK signaling pathway, typically through mutations in KRAS, BRAF, or NRAS. In a large cohort of Hungarian patients, Vereczkey et al. found KRAS mutations in 42% of LGSOC tumors and BRAF V600E mutations in 5%, confirming MAPK pathway activation as a central therapeutic vulnerability in this subtype [26]. These mutations render the tumor partially dependent on MEK signaling, making MEKi a logical therapeutic avenue. Notably, trametinib has demonstrated clinical benefit over standard care in the GOG 281/LOGS trial, improving progression-free survival in LGSOC patients. However, the therapeutic success of MEKi is mitigated by the emergence of resistance. Mechanistically, resistance has been associated with NOTCH pathway activation and loss of negative regulators such as SHOC2, prompting exploration of dual inhibition strategies combining MEKi with pan-RAF inhibitors [25]. These findings underscore the dynamic plasticity of MAPK signaling and provide a rationale for early incorporation of combination regimens in clinical trials.

Hormonal and Alternative Pathway-Directed Therapies

Apart from the MAPK pathway, hormonal therapy has been proposed as a rational treatment modality due to the presence of estrogen and progesterone receptors (ER/PR) in LGSOC [27]. Hollis et al. estimated that low PR expression is linked with poor survival, with possible implications for patient stratification for endocrine approaches. Additionally, letrozole, another aromatase inhibitor, has been shown to be effective in specific cases, including recurrent or low-burden disease [28]. Retrospective comparisons further suggest that endocrine therapy may achieve disease stabilization with lower toxicity, making it an attractive option for selected patients.

In addition to these standbys, new candidates are on the horizon. ATR inhibitors are being studied, with an emphasis on tumors -including those with replication stress or homologous recombination deficiency. Although LGSOC is considered to be genomically stable, Bradbury et al. suggest that ATR and PARP inhibitor combination approaches may be applicable in select subgroups, particularly those with DDR pathway alterations [27]. Such strategies highlight the need to integrate genomic testing beyond MAPK mutations to identify hidden DNA-repair vulnerabilities.

Epigenetic and DNA Damage Response Targets

Epigenetic therapies also show potential. Stewart et al. showed that decitabine, an inhibitor of DNA methyltransferase, triggers apoptosis in KRAS-mutated LGSOC cell lines and synergizes with BCL-2 inhibitors such as navitoclax [29]. This serves to support a biomarker-based treatment strategy where KRAS status informs the choice of therapy. Concurrently, research conducted by Corkery and others (2012, 2011) has demonstrated similar findings among synthetic cannabinoids. PRP4K was identified as a regulator of chemotherapy sensitivity through the modulation of HER2, thus suggesting a role for HER2-related pathways in the development of taxane resistance [30]. Although preclinical, these data broaden the spectrum of actionable targets beyond canonical MAPK components.

Other attractive therapeutic targets are histone methylation and transcriptional regulation, with studies that have delineated the reliance factors in LGSOC epigenetics. Wong et al. detected overexpression of C-MYC and survivin (BIRC5) in LGSOC, suggesting that both could be targets for treatment with small-molecule inhibitors or antisense oligonucleotides. Moreover, defects in chromatin remodeling complexes and cell cycle checkpoints (e.g., amplification of Cyclin E) also support the rationale for targeting CDK2/4 in a combination regimen [31].

 From a clinical viewpoint, patient-derived xenograft (PDX) and organoid models are becoming steadily more commonly used to screen drug cocktails in vitro. For example, Moujaber et al. showed that single AKT or mTOR inhibitors (even more weakly) might sensitize LGSOC cells to MEKi or endocrine therapies in combination [32]. These data support the use of rationally designed combination trials.

Hormonal Therapy Stratification

Beyond these stalwarts, however, a new golden age of exploration beckons. The gene nm23-H1 (NME1) is a candidate metastasis suppressor and is involved in the control of tumor diffusion in EOCs. Viel et al. discovered that decreased expression of nm23-H1 was associated with lymphatic metastasis, more notably in advanced serous carcinoma. They concluded that nm23 altered the propensity of metastasis even in low-grade disease. Although not traditionally targetable, its downstream signaling may be an indirect targetable vulnerability, especially when combined with mutational status [33].

Immune-Based Therapies

Finally, immune-based therapies remain largely unexplored in LGSOC, although early studies suggest that its relatively low mutational burden and immune-cold microenvironment pose challenges. Nevertheless, Hollis et al. observed immune cell exclusion and low PD-L1 expression, suggesting that immune checkpoint blockade alone is unlikely to yield meaningful clinical benefit without combination approaches [28]. Future strategies may therefore require priming the tumor microenvironment - such as with oncolytic viruses or epigenetic modifiers - to convert “cold” tumors into responsive phenotypes.

In conclusion, therapeutic innovation in LGSOC is moving decisively toward precision oncology. Interventions targeting MAPK, hormonal signaling, epigenetic regulators, and DNA damage response (DDR) are redefining treatment algorithms. Pujade-Lauraine et al. underscore that biomarker-driven trials, such as those stratifying by KRAS/BRAF mutation status or hormone receptor expression, are essential to identify patient subsets who may benefit from emerging therapies [34]. The integration of multi-omic data with functional assays in preclinical platforms will be critical to prioritize the most promising combinations and to overcome resistance mechanisms that have limited single-agent success (Table 1).

**Table 1:** Key genomic alterations and their therapeutic relevance in LGSOC.

Gene/Alteration	Frequency	Biological Role	Therapeutic Relevance	References
KRAS	~33-50%	MAPK activation	MEK inhibitors (e.g., trametinib)	[6,7]
BRAF (V600E)	5-20%	MAPK activation	May predict indolent course	[7,8,12]
NRAS	5-10%	MAPK activation	Potential MEKi target	[9,10]
NF1 (loss)	~10-15%	RAS regulation (loss = activation)	Possible sensitizer to MEKi	[11]
CDKN2A/2B deletion	~25%	Cell cycle control	Associated with aggressive progression	[6,11]
ERBB2 mutation	~6%	PI3K/MAPK activation	Potential HER2-targeted therapy	[13]
NOTCH pathway	Variable	Cell differentiation and survival	Target for combination therapy (e.g., MEKi + NOTCHi)	[11,25]
PTEN loss	~15-20%	PI3K/AKT regulation	Hormonal therapy stratification	[35]

Future directions

In light of evolving knowledge in the field of LGSOC, future directions include the establishment of personalized interventions and the incorporation of artificial intelligence (AI) analytics and genomics into clinical management.

Inherent Heterogeneity of LGSOC

The apparent inherent heterogeneity of LGSOC necessitates personalized medicine approaches based on molecular profiling and prognosis to optimize treatment strategies. This will necessitate detailed molecular annotation and prospective validation to support the translation of predictive models into clinically meaningful benefits.

Personalized Treatment Approaches

As a result, there is a new focus on molecular and protein biomarkers, which could enable providing proper prognostic and predictive tools. Yang et al. reported that these are superior to gene expression-based models for risk stratification of patients for recurrence [36]. This highlights the importance of proteomic technology as an adjunct to genomics in profiling functional changes that might evade an exclusively DNA-based profiling.

Similarly, Martins et al. demonstrated that PTEN loss is of prognostic importance and correlates with hormone receptor expression as well as with immune parameters in serous ovarian tumors. It is this genetic heterogeneity that may enable PTEN to be used to guide therapy in future trials [35]. Although not LGSOC-related, the results from Pham et al. reported the prognostic relevance of SOX2 expression in HGSOCs, providing some hope for discovering more general biological insights that may translate across histological subtypes [37]. Together, these observations demonstrate that emergent biologic targets between serous subtypes may also drive larger therapeutic approaches and further underpin histology-specific treatments.

Beltrame et al. carried out RNA sequence-based transcriptome profiling and defined distinct molecular subtypes in LGSOC based on the activation of pathways such as MAPK and PI3K. Such subtypes may be eligible for precision medicine strategies consisting of the targeting of specific pathway vulnerabilities [38].

AI and Genomic Data for Patients’ Well-Being

Applications of AI and machine learning in advancing clinical prediction and patient-centered care are on the rise today. Pan et al. emphasized the impact of multiparametric models unifying clinical, genomic, and epigenomic features to aid decision-making and reduce uncertainty in treatment pathways [39]. Moreover, Arildsen et al. presented that distinct tumor biology in LGSOC can also be captured for personalized treatment planning. Beyond contributing to improvement in care, these tools improve patient satisfaction as they empower the patient to become more involved and knowledgeable [40]. It will be important to incorporate patient-reported outcomes in these AI-enabled platforms to ensure that technological progress translates into actual improvements in quality of life.

Future directions must incorporate well-designed multi-omic strategies, biomarker-based novel therapies, and AI-led decision-support systems that aim to increase the treatment response rates and quality and experience of LGSOC care.

## Conclusions

LGSOC is a molecularly and clinically separate entity from high-grade serous carcinoma within the spectrum of EOCs. LGSOC has a relatively stable genome and a low TMB as well as substantial dependence on MAPK pathway alterations, making it quite distinct in both pathogenesis and therapeutic vulnerabilities from HGSOC. The significant contribution that KRAS, BRAF, and NRAS mutations predominate in serous borderline precursors suggests a stepwise model for tumor development and reinforces the importance of MAPK signaling in the biology of disease. Genomic, transcriptomic, and epigenomic profiling have demonstrated further layers of complexity, such as noncanonical pathway perturbations (NF1, MAP2K1), recurrent copy number alterations, and epigenetic silencing of tumor suppressor genes. These results highlight the diversity of the LGSOC and provide new targets for therapeutic investigation, especially in those that are refractory to standard therapy.

AR signaling inhibitors-testosterone production and its regulation have changed for mCRPC with the advent of abiraterone acetate and enzalutamide, as well as with other agents targeting the AR, such as apalutamide and darolutamide, and, in the metastatic hormone-sensitive space, combination abiraterone acetate and apalutamide. Targeted therapies, including MEKi, hormonal drugs, and the newer epigenetic and DNA damage response modulators, are transforming the therapeutic horizon. However, among the resistance mechanisms like NOTCH activation or compensatory signaling, rational combination strategies and strong biomarker stratification are needed. In this endeavor, patient-derived models and next-generation sequencing technologies are key. Further in the future, the combination of AI and multi-omic data may guide more personalized management and optimize prognostic instruments. Using computer models, doctors will be able to predict better how patients will respond to treatments and increase patient involvement and satisfaction. As the field of precision oncology advances, LGSOC represents a strong case for using molecular findings to inform personalized care trajectories. In summary, a comprehensive knowledge of the genomics and regulatory network of LGSOC is critical to foster creativity in therapy design, refine prognostication, and ultimately improve long-term outcomes for this special and difficult disease.
